# Assessment of Drug Activities against *Giardia* Using Hyperspectral Raman Microscopy

**DOI:** 10.3390/pathogens13050358

**Published:** 2024-04-27

**Authors:** Felicia S. Manciu, Jose Guerrero, Breanna C. Pence, Lizbeth V. Martinez Lopez, Siddhartha Das

**Affiliations:** 1 Department of Physics, University of Texas at El Paso, El Paso, TX 79968, USA; jaguerrero9@miners.utep.edu (J.G.); lmartinezl@miners.utep.edu (L.V.M.L.); 2 Border Biomedical Research Center, University of Texas at El Paso, El Paso, TX 79968, USA; 3 Department of Biological Sciences, University of Texas at El Paso, El Paso, TX 79968, USA; bcpence@miners.utep.edu

**Keywords:** Raman microscopy, giardiasis, confocal fluorescence microscopy, metronidazole, oseltamivir, drug assessment, non-tagged analytes

## Abstract

This study demonstrates the capability of Raman microscopy for detecting structural differences in *Giardia* cells exposed to different drugs and incubation times. While metronidazole (MTZ) visibly affects the cells by inducing extracellular vesicle releases of toxic iron intermediates and modified triple-bond moieties, oseltamivir (OSM) alters the phenylalanine and lipid structures. Modifications in the heme protein environment and the transformation of iron from ferric to ferrous observed for both drug treatments are more notable for MTZ. Different contents and amounts of vesicle excretion are detected for 24 h or 48 h with MTZ incubation. At a shorter drug exposure, releases of altered proteins, glycogen, and phospholipids dominate. Agglomerates of transformed iron complexes from heme proteins and multiple-bond moieties prevail at 48 h of treatment. No such vesicle releases are present in the case of OSM usage. Drug incorporations into the cells and their impact on the plasma membrane and the dynamics of lipid raft confirmed by confocal fluorescence microscopy reveal a more destructive extent by OSM, corroborating the Raman results. Raman microscopy provides a broader understanding of the multifaceted factors and mechanisms responsible for giardiasis treatment or drug resistance by enabling a label-free, simultaneous monitoring of structural changes at the cellular and molecular levels.

## 1. Introduction

*Giardia lamblia* is a flagellated protozoan parasite responsible for transmitting the waterborne enteric infection called “giardiasis”. Symptoms of giardiasis include diarrhea, vomiting, dehydration, and malabsorption. Children are especially vulnerable to giardiasis and suffer from stunted growth and decreased psychomotor development [1]. There are an estimated 200 million cases of giardiasis worldwide, with 500,000 new cases appearing each year [2]. In developing countries, the impact of giardiasis is high, with nearly 33% of individuals infected at some point in their lives [3]. In North America, about 15,579 cases were reported in 2018 [4]. A seropositivity of 55.3% was documented in Mexico [5]. However, giardiasis often goes undiagnosed and underreported [4,5,6]. Infection in a human host occurs when *Giardia* cysts are transmitted via contaminated water and food. The excystation (transformation of cysts to trophozoites) initiates in the stomach and continues in the upper part of the duodenum [7,8]. The newly differentiated trophozoites (excyzoites) attach to and replicate in the upper small intestine below the bile duct, producing infection. With the flow of bile and other intestinal milieu, the trophozoites migrate to the lower small intestine, transform back into cysts via the encystation, and are released from the host, spreading the infection [8,9]. 

Untreated giardiasis can cause serious long-term effects and illnesses, such as irritable bowel syndrome, fatigue syndrome, and food allergies, which can become chronic and affect especially individuals with weakened immune systems. The molecular mechanisms by which *Giardia*, a non-invasive intestinal parasite, causes diseases and its acquired drug resistance are still subjects of interest that require investigation [10,11,12,13,14,15,16]. While in the 1960s, metronidazole (MTZ) and other nitroimidazoles were the most commonly prescribed treatment for giardiasis [11,12,13], the increase in the recently reported resistance to these drugs worldwide is of concern [14]. Complications arise because this resistance is multifactorial and complex, with known mechanisms of action involving common pathways such as oxidoreductases, oxidative stress responses, and DNA repair proteins [15,16]. Furthermore, *Giardia*’s tetraploid nature makes it difficult to identify genetic markers of resistance [17]. 

Despite the numerous targeted molecules that MTZ affects, their relative importance in protozoa destruction by the drug remains uncertain [18]. An active mechanism is the activation of MTZ by partial reduction upon its passive diffusion into the cell. This process can be accomplished by either oxidoreductases and/or nitroreductases of various classes, such as pyruvate ferredoxin oxidoreductases [19]. The fact that oxidoreductases are often mutated or differentially expressed in MTZ-resistant pathogens brings additional hindrances [20,21,22], making the exact structure of the MTZ’s toxic radical or site of damage diverse. What has been established is the MTZ’s role in damaging the pathogen’s common upregulated general repair mechanisms to oxidative stress response and DNA synthesis halt [19]. Also, it has been demonstrated that the drug’s selectivity for anaerobic or microaerophilic cells is a consequence of its active form converting rapidly to the unreduced prodrug one in the presence of oxygen [23]. Among the non-metronidazole classes of compounds, several repurposed drugs such as auranofin, fumagillin, orlistat, omeprazole, and disulfiram [24], and synthetic analogs of triazoxins [25] and phosphonoxins [26] have shown promising activity against the *Giardia* parasite in culture. However, all these compounds either have multiple targets or modes of action that need to be determined.

Currently, *Giardia* protozoa methods of detection rely on microscopic visual findings of the cyst or trophozoite in stool samples (ova and parasite examination), as well as immunological methods [27]. A standard fluorescence microscopic analysis is needed for further confirmation of the immunological testing results. These detection methods have some shortfalls, including being time-consuming and using different antibodies depending on the specificity in the case of immunological tests. In addition, these tagging substances could react with different targets other than the protozoan of interest. Consequently, several false positive results could arise, triggering an unnecessary additional analysis of samples for other health concerns. Other reports consist of immunological tests that failed to detect the pathogen in contaminated water samples, as a result of the antibody reacting with only typical variants of a protozoan [27]. Lastly, current immunological tests cannot differentiate between infected or viable protozoan cysts (or oocysts) and uninfected ones. Imaging of morphological alterations of the parasite exposed to drugs by transmission electron microscopy, although providing such information with a very high resolution, is expensive and not always available [28]. For these reasons, finding alternative methods of investigation of these anaerobic protozoa and treatments with non-nitroimidazole-based drugs is essential. These new, unconventional methods of investigation need to have the capability of detecting with high accuracy and confirming at the molecular level the processes involved in *Giardia*’s resistance.

Raman microscopy is a non-invasive imaging technique that combines standard Raman spectroscopy with optical imaging and allows for in situ measurements of non-tagged analytes [29,30,31,32,33,34,35,36,37]. While Raman spectroscopy has its advantages in identifying the structural changes of compounds or metabolites, especially those containing light atoms, oxygen, sulfur, nitrogen, and carbon that can form double and triple bonds with distinct vibrational features, Raman microscopy by enabling the recording of thousands of spectra can uniquely resolve and simultaneously capture all variations accurately. Raman microscopy has been used in detecting the structural contents of biomolecules and their alterations in a variety of technological applications directed at identifying different types of viruses, bacteria, eukaryotic cells, and cancer [29,30,31,32,33,34,35,36,37]. It benefits from the advantages of being fast, with microsecond acquisition time per spectrum, being not sensitive to strong water absorption, and providing well-defined features narrower than detected in standard fluorescence microscopy. While these advantages allow for an improved signal collection, it is of importance that it does not require additional labeling of samples by external agents and dyes. This is a significant advantage for identifying directly, accurately, and quickly biomolecular changes occurring in cells’ biochemical compositions.

In our previous work, we found that the FDA-approved antiviral compound oseltamivir (OSM) or Tamiflu^®^ disassembled giardial lipid rafts (gLRs) [17] and reduced the release of exosome-like small vesicles significantly [38]. A proteomic analysis revealed that the disruption of gLRs using OSM reduced the overall number of virulence factors present in exosomes. It also reduced giardial infection in mice, supporting the importance of gLRs in host–parasite interactions. It is known that gLRs and exosome assembly are interconnected and play a crucial role in the ability of the parasite to attach to the host intestinal epithelium, produce infection, and modulate the host cell’s response to infection.

In this study, we use confocal Raman microscopy as a new modality for label-free detecting and imaging of these protozoa with a very high resolution. In addition to analyzing the biochemical composition of the untreated cells, investigations of the factors and mechanisms responsible for the structural changes occurring in the MTZ- and OSM-treated cells are presented comparatively here. Such an analysis could open new ways of examining giardiasis by complementary methods and monitoring drugs’ efficacies.

## 2. Materials and Methods

### 2.1. Sample Preparation

*Giardia lamblia* assemblage of trophozoites (strain WB C6; ATCC No. 30957) was cultured in modified Diamond’s TYI-S-33 medium supplemented with 5% heat-inactivated adult bovine bile, 0.5 mg/mL bovine bile, and 100 µg/mL piperacillin following standard procedures [39,40]. The trophozoites were cultured at 37 °C in screw-cap glass tubes until an 80–90% confluence was reached and divided routinely every 3–4 days.

The following protocol was employed for the cell treatment with the drugs. Stock solutions of powder MTZ (Sigma-Aldrich, St. Louis, MO, USA) and OSM (Selleckchem, Houston, TX, USA) diluted in dimethylsulfoxide solution (DMSO; Sigma-Aldrich, St. Louis, MO, USA) were used to achieve 1 µM and 20 µM drug concentrations, respectively. Next, the trophozoites were incubated with the compounds for 24 h or 48 h in T25 flasks filled with TYI-S-33 medium, and the parasites were harvested by centrifugation (1500× *g* for 5 min), followed by washing in PBS. The samples were fixed with 4% paraformaldehyde solution (Sigma-Aldrich, St. Louis, MO, USA) for 10 min at room temperature and washed in PBS followed by additional washing in double-distilled water. Approximately 1 × 10^4^ cells in a volume of 30 µL were dropped-cast onto a coverslip and allowed to air-dry before the confocal Raman microscopic analysis. To investigate the cells’ membranes and lipid rafts using confocal fluorescence microscopy, the fixed samples were exposed to cholera toxin subunit B (CTXB) conjugated to Alexa fluor™ 488 (1:1000) (Invitrogen, Carlsbad, CA, USA) for 10 min at 4 °C, followed by washing in PBS. The cells immunostained with CTXB (1:200 dilution) in 1% normal goat serum (NGS) for 15 min at 4 °C were three times washed with PBS and resuspended in DAPI solution (1 µg/mL) for 15 min. The excess DAPI stain was washed in PBS. The cells were mounted on glass slides with Vectashield Antifade Mounting medium (Vector Laboratories, Newark, CA, USA).

### 2.2. Instruments

An alpha 300RAS WITec confocal Raman system (WITec GmbH, Ulm, Germany) was employed to acquire the surface mapping images of untreated and drug-treated *Giardia* cells. The system was equipped with a microscope coupled to a triple grating monochromator/spectrograph via an optical fiber of 50 μm core diameter. A 532 nm light excitation of a frequency-doubled neodymium-doped yttrium–aluminum–garnet (Nd:YAG) laser was used at a low output of less than 3 mW to avoid sample photodegradation. Data acquisitions were performed with a 50X air objective lens (Nikon, Tokyo, Japan) of 0.75 numerical aperture (NA) and a thermoelectrically cooled Marconi CCD camera. The WITec Control software that enables piezoelectric stage control for sample scanning was employed for fast spectral recording at an integration time of 50 ms per spectrum. Arrays of 100 × 100 Raman spectra were collected for the surface Raman mapping images of 50 μm × 50 μm scan sizes for multiple cells’ images and 20 μm × 20 μm scan sizes for single-cell images.

For a confocal microscopy analysis of fluorescently labeled *Giardia* samples, a Carl Zeiss LSM 700 (Zeiss, Oberkochen, Germany) equipped with a 63X oil Plan-Apochromat Zeiss objective lens and two laser excitations of 488 nm (set to level 5 and 946 gain) and 405 nm (set to level 10 and 930 gain) were utilized. Image capture and analysis were performed using the Zen 2009 software (Zeiss, Oberkochen, Germany). Images were captured as Z-stacks, with five slices at an interval of 1.17 µM and 1024 pixels per image, and processed using the maximum intensity projection. Approximately 20 cells per field were assessed for CTXB labeling, using eight fields for measuring the mean fluorescence intensity and quantifying the number of positive cells to CTXB labeling. Statistics were carried out using a one-way ANOVA with GraphPad Prism 10.

## 3. Results and Discussion

**Figure 1:**
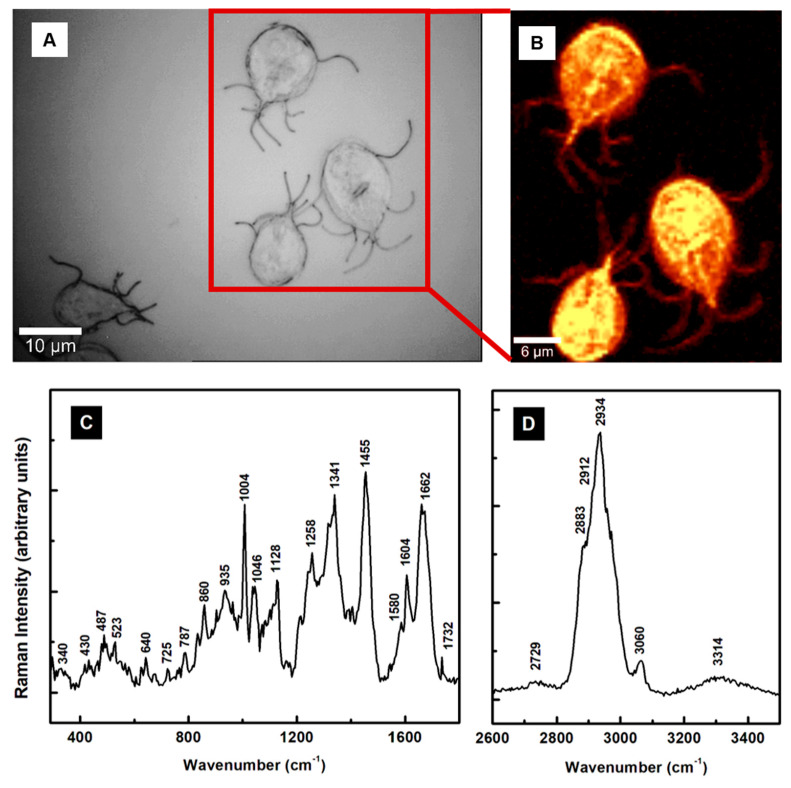
(**A**,**B**) Optical image and the corresponding surface Raman mapping image of multiple *Giardia* cells. A bright yellow pseudo-color corresponds to a higher Raman intensity. (**C**,**D**) Integrated Raman spectrum associated with the confocal Raman image for lower and higher vibrational regions, respectively.

Since *Giardia lamblia* has been investigated extensively, the scarcity of its spectroscopic studies is surprising. Only a couple of its Raman analyses have been reported [30,31]. In one study, the parasite’s ability to encode several putative heme-biding proteins was investigated [30], and in another its detection in contaminated water by surface-enhanced Raman spectroscopy [31]. The cysts of this protozoon were immunogold-labeled in the second study. To fill this gap and take advantage of the label-free Raman microscopy in finding accurate details of the biochemical structure of *Giardia* cells, we present in Figure 1A–D the images acquired optically and using surface confocal Raman mapping, together with the associated spectroscopic measurements. No additional functionalized nanoparticles, fluorophores, or antibodies were used as tagging.

The optical image in Figure 1A recorded with a white-lamp source is for visualizing the specific micro-regions of acquiring the surface confocal Raman image shown in Figure 1B. The hyperspectral resolution employed in collecting the confocal Raman image facilitates observation of the flagella beside the characteristic teardrop body shapes of the cells. A comprehensive understanding of the bio-structural composition of the cells entails a spectroscopic analysis of the observed Raman vibrational lines. Thus, the integrated spectrum that is the average of about 10,000 independently acquired spectra in different locations of the sample is presented in Figure 1C,D, in the lower and higher vibrational regions, respectively. Using such an average for an image containing more than one cell accounts for the intrinsic non-uniformity of any sample and the expected spectral variations from cell to cell. Considering that the majority of the biological cells have a featureless, silent Raman region from 1800 to 2600 cm^−1^, this region is not incorporated. The frequencies of the observed vibrational lines corresponding to different cell constituents and their tentative assignments are summarized in Table 1.

The fingerprint vibrational region for physiological and biochemical information presented in Figure 1C reveals many sharp lines. In addition to the dominant peaks associated with lipids and proteins at 1004, 1046, 1128, 1258, 1341, 1455, 1064, and 1662 cm^−1^, weak spectral features attributed to heme-binding proteins, glycogen, cysteine, phospholipids, and nucleic acids are observed at 340, 430, 487, 523, 640, 674, 725, and 787 cm^−1^ lower frequencies [29,30,32,41,42,43]. Of notice is that *Giardia*, which lacks mitochondria, is the only eukaryote containing at least five heme-binding proteins, with four belonging to the cytochrome b_5_ family (electron-transfer proteins that enable incorporation of heme) and one to the flavohemoglobin class. As specified in Table 1, some of these vibrations are assigned tentatively to such proteins.

Intense, convoluted lines at 2883, 2912, and 2934 cm^−1^ related to the CH_2_ and CH_3_ vibrations of lipid and protein contents dominate the Raman spectrum in the high-frequency region [29,32]. Other less intense and broad features are at 2729 cm^−1^ (–CH stretching), 3060 cm^−1^ (=CH of unsaturated fatty acids), and 3314 cm^−1^ (–OH of water).

**Figure 2:**
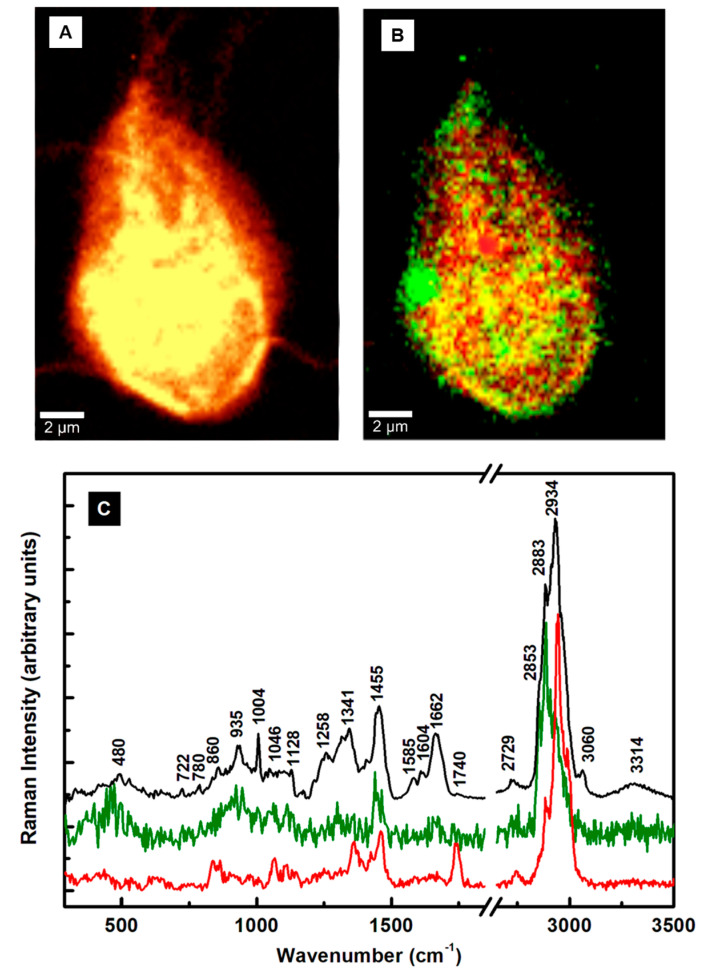
(**A**,**B**) Confocal Raman mapping images of a *Giardia* cell performed by considering all constituents and by selectively filtering the lipids (green pseudo-color) and proteins (red pseudo-color), respectively. (**C**) Raman spectra associated with each image. The integrated spectrum (black line) is associated with the image (**A**). The same color code is maintained between the image (**B**) and the spectra of lipids (green pseudo-color) or proteins (red pseudo-color).

Figure 2A shows a representative confocal Raman image of a single cell selected from more than seven images of independent cell mapping acquired at an even higher spectral resolution. The bright yellow corresponds to a stronger Raman signal. While all cell’s biocomponents were considered indiscriminately in acquiring this image, a selective identification of lipids (green pseudo-color) and proteins (red pseudo-color) was performed for the confocal Raman mapping in Figure 2B by filtering out the bands centered at about 2883 cm^−1^ and 2934 cm^−1^, respectively. In addition to a random distribution of these constituents throughout the cell body, their accumulations are also observable in this image. An example is the predominant lipid content (green pseudo-color) at the cell’s boundary. The yellow color in Figure 2B is due to the combination of red and green pseudo-colors, confirming overlapping between them at numerous locations. The Raman spectra associated with these images in Figure 2C validate a protein- and lipid-dominant composition. In support of this remark and for easier correspondence with constituents, the same pseudo-color code is maintained in these spectra and the image in Figure 2B. The black line spectrum in Figure 2C corresponds to the image in Figure 2A and is similar to the spectrum presented in Figure 1C,D for multiple cells. The subtle differences in the intensities of the various structural signature peaks arise from less content imaged from a single cell. While imaging at a higher resolution has the advantage of precisely distinguishing the locations of components and their potential overlapping, it hampers the observation of the inherent variability from cell-to-cell configurations. Usually, statistical analyses or machine learning approaches that enable the depiction of elusive changes are employed to identify this variability. Since each of these integrated spectra (black line in Figure 1C,D and Figure 2C) consists of averaging 10,000 independently acquired spectra during the confocal Raman imaging at every pixel, not only their similarity but also the powerfulness of this new modality of analysis with significant accuracy are revealed. The standard error of such media is inversely proportional to the square root of the sampling points and is vanishingly minor (a point error bar). Appropriate spectral background subtraction that includes the broad bands of the glass substrate, vertical translation, and exclusion of the featureless frequency region from 1900 to 2600 cm^−1^ were employed. 

At high frequencies, the distinct separation observed between the most intense vibrations at 2883 cm^−1^ for lipids and 2934 cm^−1^ for proteins agrees with some irregular aggregation of these constituents in Figure 2B. Their random and overlapping distributions associated with the yellow color are more noticeable at lower frequencies, with contributions from both lipids and proteins at 1128, 1341, and 1455 cm^−1^.

**Figure 3:**
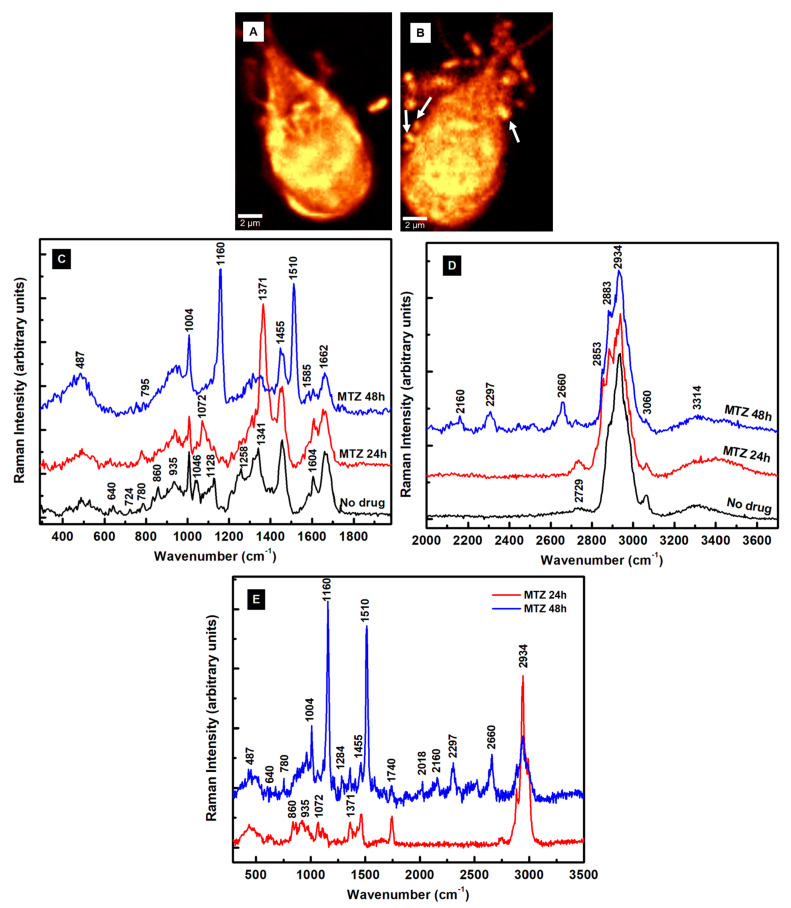
(**A**,**B**) Confocal Raman mapping images of *Giardia* cells treated with 1 µM MTZ for 24 h and 48 h, respectively. A visible membrane blebbing is seen for the cell’s 48 h incubation time with MTZ and marked with white arrows. (**C**,**D**) Integrated Raman spectra associated with image (**A**) (red line spectrum) and image (**B**) (blue line spectrum) in the low-frequency and high-frequency regions, respectively. The integrated spectrum (black line) of untreated cells is also plotted for reference. (**E**) The Raman spectra associated with the microdroplets observed in images (**A**,**B**), as labeled (i.e., red line spectrum for 24 h of treatment and blue line spectrum for 48 h of treatment).

To understand the action of the drugs by using Raman microscopy, a comparison between the untreated and MTZ-treated cells for 24 h and 48 h is presented first in Figure 3A–E. While the shape of the trophozoite in Figure 3A seems generally unaffected after a 24 h of treatment, the parasite’s appearance changes obviously in Figure 3B after a 48 h incubation time with MTZ. In addition to the membrane damage, this image reveals extracellular vesicles surrounding the cell. The single microdroplet observed in Figure 3A for 24 h of treatment implies more efficacy of the drug with time. Membrane blebbing marked with white arrows in Figure 3B and peripheral vesicle releases are common phenotypes of the drug’s influence. 

The corresponding integrated Raman spectra presented in Figure 3C,D, with red and blue lines for 24 h and 48 h of treatment, respectively, confirm these dramatic morphological modifications due to the MTZ administration. A normalization to the most intense Raman vibrational line at 2934 cm^−1^ is applied to the spectra besides background subtraction and vertical translation. The reason for this normalization is to eliminate potential laser power variations between measurements. The Raman spectrum of untreated cells (black line) is included for reference. Additional information after the MTZ treatment that concerns cell variability is provided in Figure S1 in the Supplementary Materials section. 

A visible increase in the intensity of the Raman vibrational line at 1371 cm^−1^ is seen after 24 h of drug treatment (red line spectrum). This line shifts from its original location at 1341 cm^−1^ (C–N breathing mode) for the untreated cells and is associated tentatively with oxidation of the heme Fe(III)-binding proteins [41]. In Raman spectroscopy, the most intense bands of such proteins arise from the prosthetic group of the b cytochromes and contributions of protoporphyrin ring π to π^*^ transitions. They do not necessarily directly involve the central iron atom, but its ligation and oxidation state. Biological porphyrins have hydrogen atoms bound to the various carbon atoms of the methide or outer pyrrole moieties, with at least one of the axial ligands usually from the partially protonated imidazole side chain of histidine. Another piece of supporting evidence of the heme oxidation process is the slight increase of the 2729 cm^−1^ feature (see Figure 3D), which is a bend overtone of the 1341 cm^−1^ line [41]. 

The observed intensity increase of the 780 cm^−1^ peak corresponding to denatured nucleic acids could underline the MTZ’s cytotoxic role in modifying DNA synthesis [15]. A similar alteration of nucleic acids was observed in drug-resistant cancer cells [34,35]. Currently, the anaerobic metabolic pathway of drug activation consists of its partial reduction through the nitro group. Within the trophozoites, ferredoxins (electron transport proteins) activate the drug by donating electrons to the MTZ’s nitro group and creating a gradient that favors its intracellular transport. The electron-acceptor-reduced MTZ binds covalently to DNA, impairing its structure and further unaltered synthesis. Consequently, in addition to improper cell functioning, it contributes to cell death as the ultimate result. Signatures of a metabolic glycolytic pathway, which involves pyruvate:ferredoxin oxidoreductase (PFOR) enzymes, could contribute to the disappearances of the vibrational lines at 860 and 1046 cm^−1^. However, the appearance of a new peak at 1072 cm^−1^ attributed to lysine suggests another way of the parasite’s protection from nitrosative stress and adaptability for survival, potentially through a histone lysine acetylation or a new histone post-translational protein modification [29,44]. Other bio-structural changes involving the lipid content are the visible intensity reductions of characteristic vibrations at 1128 and 1258 cm^−1^ in Figure 3C and the increases in the well-defined peaks at 2853 and 2883 cm^−1^ in Figure 3D. These latter high-frequency vibrational changes could also arise from the effect of the DMSO solvent used for drug dilution, as revealed in Figure S2 in the Supplementary Materials section.

The investigation of the cells after 48 h of incubation (blue line spectrum) shows further modifications in the cell biostructure, with new intense peaks at 1160 and 1510 cm^−1^, observed for the first time in this study. Tentative assignments for these peaks are either to a change in the iron complex oxygenated state ligation and strong activation of the carbon monoxide-bound complex of ferrous heme, Fe^II^CO, or to deoxyadenosine triphosphate (dATP) structural changes [32,41,42]. In an almost apoptotic state after 48 h of MTZ treatment, the effects of a different iron coordination/oxidation and alteration of the heme protein’s structure are also seen in the intensity decrease of the 1341 cm^−1^ vibration, which might correspond to a reduced form of the cytochrome P-450 and cysteine ligands’ strong π-donor properties [41,42]. The change in the iron oxidation state from ferric to ferrous corroborates with a decreased activity in the pyruvate:ferredoxin oxidoreductase that is required for further MTZ’s activation, and, potentially, with *Giardia*’s acquired resistance. The second tentative assignment for these lines to *dATP*-activated lysates correlates with the cell respiratory chain mechanism that, if broken, could drive the cell from an apoptotic transition to death. It also correlates with the cell’s membrane damage. Other intensity increases are seen for the broad bands at 487 cm^−1^ (glycogen or ferrous compound) and 935 cm^−1^ (deoxygenated heme cell porphyrin). The absence of the 860 and 1046 cm^−1^ vibrational lines also confirms a broken glycolysis repair mechanism in these poorly oxygenated *Giardia* cells. The very weak presence of the 780 cm^−1^ band and the new feature at 795 cm^−1^ demonstrate the effects that alter the DNA structure. A similar observation is for the Raman lines at 1585 and 1604 cm^−1^, suggesting an additional occurrence of an NDHA oxidase process. Lastly, a potential correlation between the 1160 cm^−1^ new feature and that of adenine at 1150 cm^−1^ or cytosine NH_2_ rocking and C–N stretching at 1196 cm^−1^ infers another protozoa mechanism of protection through releases of transformed moieties [29]. The proximity of this line to the peak at 1175 cm^−1^ observed in the immunogold-labeled *Giardia* cyst samples used for identifying the parasite also implies its potential need for encystation as a defense [31].

Further structural changes are observed in the standardly silent high-frequency region, with new Raman features at 2160 cm^−1^ (–C≡C), 2297 cm^−1^ (–C≡N), and 2660 cm^−1^. They could be associated with the presence of alkylated and nitrated lipid raft moieties due to the dramatic damage of the sphingomyelin and cholesterol-rich domains in the cell membrane [43,45]. There are no such triple-bond vibrations in the cases of untreated and 24 h-treated cells. A compositional analysis of the extracellular vesicles seen in Figure 3A,B is presented in Figure 3E. The red line spectrum corresponds to the average of spectra recorded in the location of the microdroplets in Figure 3A, and the blue line spectrum for all microdroplets in Figure 3B, including those from membrane blebbing. These spectra show a startling difference in their bio-composition. While for the 24 h-treated cells, a release of altered proteins, glycogen, and phospholipids dominates, agglomerates of modified iron complexes from heme proteins and triple-bond moieties are the main contributors in the case of 48 h treated cells.

**Figure 4:**
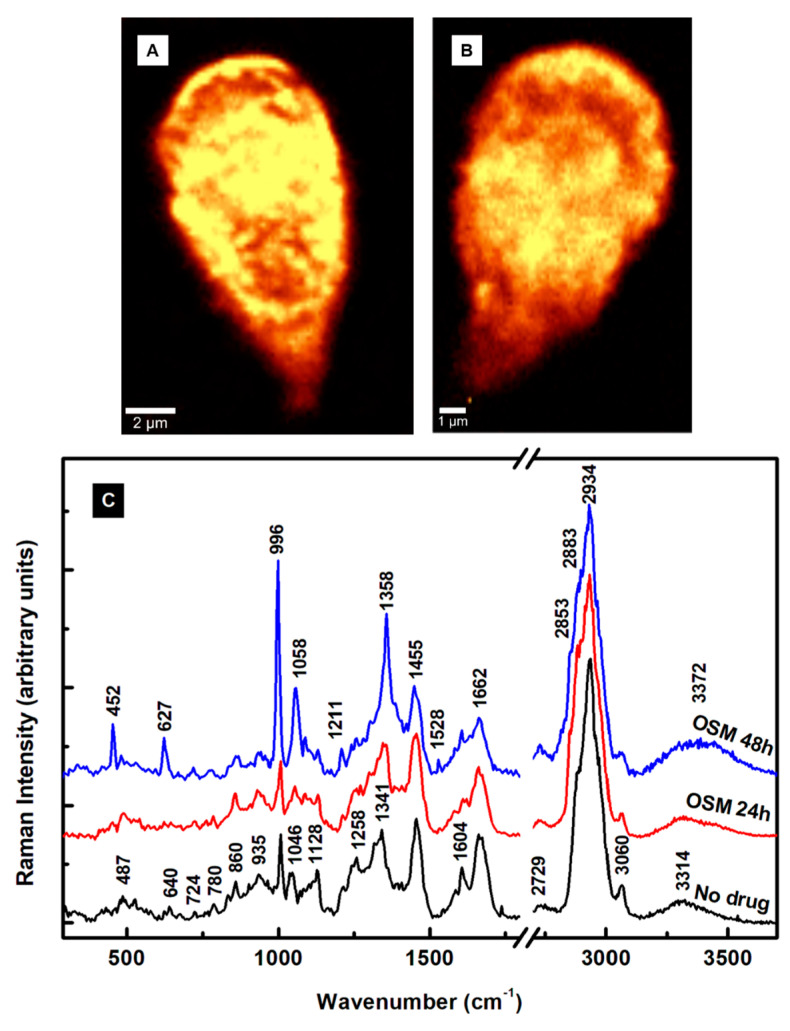
(**A**,**B**) Confocal Raman mapping images of *Giardia* cells treated with 20 µM OSM for 24 h and 48 h, respectively. Membrane damage and cell size decrease (scale bar of 1 μm) are observed for the cell after a 48 h incubation time with OSM. (**C**) Integrated Raman spectra associated with image (**A**) (red line spectrum), image (**B**) (blue line spectrum), and the control untreated cells (black line spectrum).

The outcomes of the OSM’s effect on the *Giardia* cells are presented in Figure 4A–C. The first significant remark is the absence of extracellular vesicles in Figure 4A,B, which correspond to confocal Raman images of cells after 24 h and 48 h incubation time with OSM, respectively. This remark indicates a potentially different mechanism of action of the OSM compared to that of the MTZ. A similarity between the two drug treatments is the damage to the cell’s membrane observed in both Figure 3B and Figure 4B for extended drug use. A much smaller size for the cells exposed to OSM for 48 h is seen in Figure 4B, as indicated by the 1 µm scale bar. Again, the integrated spectra presented in Figure 4C are subject to background subtraction, vertical translation, normalization to the most intense Raman vibrational line at 2934 cm^−1^, and exclusion of the frequency region from 1900 to 2600 cm^−1^. The Raman spectrum of 24 h treatment with OSM (red line) shows a few vibrational changes compared with the spectrum of untreated cells (black line). The obvious ones are the slight intensity decreases of the phenylalanine peak at 1004 cm^−1^ and 1604 cm^−1^, with the latter having contributions from tyrosine as well. The phenylalanine differs structurally from tyrosine only by a hydroxyl group of the benzene ring. Since both vibrations relate to phenylalanine, we assume that the drug probably affects it more. Other noticeable decreases are in the intensities of the vibrations corresponding to lipids and phospholipids at 1128 and 1258 cm^−1^. This observation agrees with our previously reported effect of the drug on giardial lipid rafts, such as the reduced release of exosome-like small vesicles [17]. The slight increase in the intensity of the heme protein line at 1341 cm^−1^ and its shift to 1350 cm^−1^ demonstrate that OSM acts on this essential constituent, too. Considerable modifications attributed to these constituents appear in the spectrum of the cells treated with the drug for 48 h. For example, the intense peak at 996 cm^−1^ defines a strongly altered phenylalanine, and the vibrational line at 1058 cm^−1^ can be associated again with histone lysine acetylation or a new histone post-translational protein modification [29,44]. In addition, the peaks at 452, 627, and 1358 cm^−1^ reveal visible heme protein and lipid structural variations.

Oseltamivir treatment is for alleviating seasonal influenza symptoms and a dysfunctional immune system. One consequence of a weakened immune system is an altered downregulation of phenylalanine hydroxylase (PAH) activity. This remark explains and substantiates the degree of changes seen in the primary structures of phenylalanine, lipids, heme, and non-heme proteins of *Giardia* cells upon exposure to the drug. The non-heme iron-containing monooxygenase PAH is essential to catalyze the oxidation of phenylalanine to tyrosine. The catabolism of phenylalanine and its lack of hydroxylation to tyrosine after 24 h treatment with OSM (see the red line spectrum in Figure 4C) confirm a broken homeostasis of this amino acid. In addition, to allow phenylalanine conversion to tyrosine, the catalytic mechanism of PAH involves the reduction of iron from ferric (III) to ferrous (II), as underlined by the intensity and frequency changes of the line at 1341 cm^−1^ with treatment. These observations are more evident after 48 h of treatment with the drug, where the unstabilized PAH activity triggers significant conformational changes in the phenylalanine and heme proteins at 996 cm^−1^ and 1358 cm^−1^, respectively. To ensure that these observations are valid even for the inherent cell variability, we provide in Figure S3 in the Supplementary Material section additional integrated Raman spectra associated with different cell mapping images.

**Figure 5:**
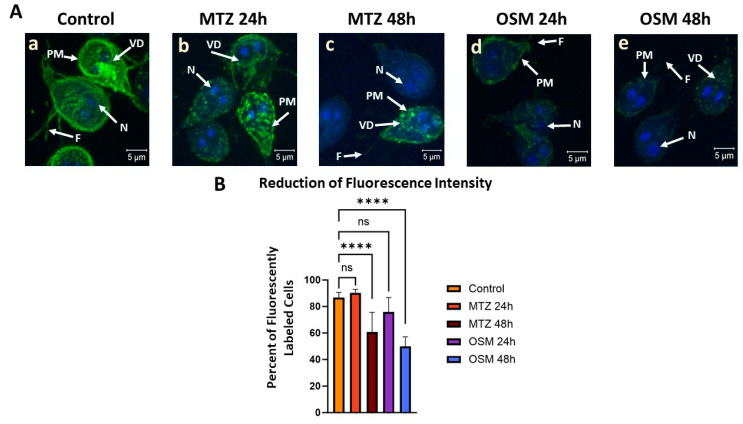
Confocal fluorescent microscopy of *Giardia* trophozoites after treatment with 1 µM MTZ or 20 µM OSM for 24 and 48 h. (**A**): Trophozoites labeled with Alexa 488-conjugated cholera toxin subunit B (CTXB) and with DAPI for lipid rafts (green) and nuclei (blue) imaging, respectively. Both MTZ and OSM treatments show a reduction in lipid raft labeling by less intense and punctate CTXB labeling pattern, with OSM 48 h treatment having the most impact (panel **e**). (**B**): The number of trophozoites positive for CTXB labeling shown as the percentage of fluorescently labeled cells. Approximately twenty cells per field were assessed for CTXB labeling, with eight fields being utilized. The significant reduction of cells positive for CTXB labeling, observed for 48 h of MTZ and OSM treatments, can be associated with a decreased lipid raft assembly. A greater impact is seen for OSM administration compared to MTZ at each respective time point. Legend: PM: plasma membrane; VD: ventral disc; N: nucleus; F: flagella; ****: *p* < 0.0001; ns: non-significant.

Supporting evidence using fluorescent microscopy of the MTZ and OSM incorporations into the cells and their impact on the plasma membrane, flagella, and ventral disc structures are shown in Figure 5A. Fluorescently conjugating the trophozoites with CTXB, a known label of GM1 ganglioside present in raft domains, besides enabling visualization of the overall cell, targets the dynamic gLR assembly [17]. 

The intensity reduction of the labeling from the untreated cells (panel a) to a dotted or punctate-like pattern after treatment with MTZ at both 24 h and 48 h (panels b and c) demonstrates alterations of these structures. A more drastic effect in reducing visible labeling is observed for the 48 h incubation with MTZ. These remarks are in agreement with our previous observations from a Raman analysis, with less presence of altered lipids and proteins and more prevailing iron-sulfur metabolites at longer drug usage.

Treatment with OSM further reduces CTXB labeling at both 24 h and 48 h (panels d and e), with rafts being harder to visualize than in the case of MTZ treatment. Again, this effect is more apparent in the 48 h of OSM administration, yielding trophozoites with gLR domains almost invisible to CTXB labeling. A quantification of trophozoites for CTXB labeling presented in Figure 5B confirms a significant decrease in the number of cells positive for CTXB labeling for treatment with each drug. The greatest raft labeling fluorescence decrease is for OSM, with only about 50% of the trophozoites showing visible CTXB labeling. This observation implies that OSM strongly affects the assembly of GM1-containing gLRs.

## 4. Conclusions

While the molecular mechanisms by which *Giardia* causes diseases have been investigated widely, the multifaceted factors involved in the acquired resistance of this intestinal parasite to drugs remain of significant research interest. Testing new drugs and using new methods of investigation, such as the ones presented here, enable a broader understanding of these aspects and facilitate new findings through a label-free, faster, and more accurate way of detecting and monitoring structural changes at the molecular level. The drug concentrations, corresponding to the IC_50_ toxicological limits, are below the Raman capability limit of their direct detection. This limitation does not preclude confirming their influence on the cell constituents, as this study’s outcomes demonstrate.

An investigation of the cells exposed to the commonly prescribed MTZ for *Giardia* treatment validates that prolonged drug usage is more beneficial and reveals ferredoxins’ importance in activating the drug. More importantly, it validates the diversity of potential mechanisms of an intracellular drug’s action on different constituents. An induced extracellular release of toxic intermediates of different molecular compositions for 24 h or 48 h of treatment is observed. Another dominant effect is the contribution of heme-like proteins and their structural alterations that impede proper cell metabolic functioning. The heme oxidation process and change in the environment and coordination/oxidation of its central iron atom are an unexplored potential way of cell protection and/or acquired resistance to the drug. We tentatively assigned the new and intense vibrational lines observed for the first time in this work to this process. The supporting evidence is the release of altered triple-bond moieties and iron complex metabolites in the case of 48 h treated cells. A treatment with MTZ for 24 h induces small excretions of proteins, glycogen, and phospholipids.

The absence of these extracellular vesicle releases is the first evidence that a different mechanism of action of the OSM compared to that of the MTZ occurs. The spectroscopic analysis shows that this drug alters phenylalanine and lipid structures dominantly, substantiating its usage intent for alleviating seasonal influenza symptoms and a dysfunctional immune system. It is known that an altered downregulation of PAH activity happens because of a weakened immune system, which, in *Giardia* cells, triggers significant conformational changes in phenylalanine and heme proteins. In addition to the relatively similar modifications observed in the vibrational lines of heme and non-heme proteins, damages to the cells’ membranes are seen for both drugs’ administrations at 48 h. The drug incorporations into the cells and their impact on the plasma membrane and the dynamics of lipid raft confirmed by confocal fluorescence microscopy demonstrate a more destructive extent by OSM, corroborating the Raman results.

This is the first study where confocal Raman microscopy was employed as a new modality for label-free detecting and imaging of these protozoa and analyzing the changes in the biochemical composition of cells due to drug administration. This analysis unlocks new findings in monitoring drugs’ efficacies and understanding potential ways of overcoming giardiasis resistance to treatment.

## Figures and Tables

**Table 1:** Raman vibrational bands and their assignments with tentative attributions.

Raman Shift (cm^−1^)	Assignment	Attribution
340	C–C–C pyrrole ring deformation	Heme-binding proteins, cytochrome b_5_ [29,30,41,42]
430	C–C–C pyrrole ring deformation	Heme-binding proteins [29,30,41,42]
487	Fe–CO, C–C stretching	Heme-binding proteins, glycogen [32,41,42]
523	Fe–CO, S–S stretching	Heme-binding proteins, cysteine [41,42]
640	C–N, C–C twisting	Heme-binding proteins, guanine, tyrosine [29,32,41,42]
725	C–N ring breathing	Phospholipid, adenine [29,32,41]
787	Pyrrole breathing	Cytosine, nucleic acids [29,30,32,41,42]
860	C–C stretching, –CH out-of-plane bending	Tryptophan, glycogen, polysaccharide [29,32]
937	–CH_3_ symmetric stretching	Lipids, proteins (α-helix) [29]
1004	Benzene ring breathing	Phenylalanine, collagen [29]
1046	C–C, C–N, C–O stretching	Lipids, proteins, glycogen [29]
1128	C–C stretching	Lipids [29]
1258	–CH_2_ deformation, N–H bending, C–N stretching	Lipids, Amide III [29,32]
1341	C–N breathing, –CH_2_ deformation	Heme proteins, lipids, adenine, guanine [29,30,41,42]
1455	C–N bending, –CH_3_ out-of-plane deformation	Lipids, proteins [29]
1580	N–H bending, C–N stretching	Tryptophan, Amide II, NADH [29]
1604	C–O stretching, C=C bending	Phenylalanine, tyrosine [29]
1662	C=O stretching, out-of-plane C–N stretching	Amide I [29]
1732	C=O stretching	Lipids, phospholipids, triacyl glycerides [29]
2729	–CH stretching	Saturated fatty acids [29]
2883	–CH_2_ antisymmetric stretching	Lipids, raft-like ordered domains [29,32,43]
2912	–CH_3_ symmetric stretching	Lipids, proteins [29,32]
2934	–CH_3_	Proteins, fat, cholesterol [29,32]
3060	=CH	Unsaturated fatty acids [29,32]
3314	–NH, –OH	Proteins, water band [29,32]

Refs: [29,30,32,41,42,43], and the references therein.

## Data Availability

The original contributions presented in the study are included in the article/supplementary material; further inquiries can be directed to the corresponding authors.

## Supplementary Materials

The following supporting information can be downloaded at: https://www.mdpi.com/article/10.3390/pathogens13050358/s1, Figure S1: Integrated Raman spectra from different cell images for the untreated and MTZ-treated for 24 h and 48 h (each spectrum averaging 10,000 spectra acquired per image); Figure S2: DMSO-treated Giardia cells.; Figure S3: Integrated Raman spectra from different cell images for the untreated and OSM-treated for 24 h and 48 h (each spectrum averaging 10,000 spectra acquired per image).
