# Supplementary material for: Assessment of Drug Activities against Giardia Using Hyperspectral Raman Microscopy
Source: Pathogens. 2024 Apr 27;13(5):358. doi: 10.3390/pathogens13050358 (PMC11124377; doi:10.3390/pathogens13050358)
Supplement: Supplementary file 1 [file pathogens-13-00358-s001.zip › pathogens-2909282-supplementary.pdf]

pathogens

MDPI

Article

Assessment of Drug Activities against *Giardia* Using Hyperspectral Raman Microscopy

Felicia S. Manciu [1,2,*], Jose Guerrero [1], Breanna C. Pence [3], Lizbeth V. Martinez Lopez [1] and Siddhartha Das [2,3,*]

1 Department of Physics, University of Texas at El Paso, El Paso, TX 79968, USA; jaguerrero9@miners.utep.edu (J.G.); lmartinezl@miners.utep.edu (L.V.M.L.)
2 Border Biomedical Research Center, University of Texas at El Paso, El Paso, TX 79968, USA
3 Department of Biological Sciences, University of Texas at El Paso, El Paso, TX 79968, USA; bcpence@miners.utep.edu
* Correspondence: fsmanciu@utep.edu (F.S.M.); sdas@utep.edu (S.D.); Tel.: +1-915-747-8472 (F.S.M.); +1-915-747-6896 (S.D.)

Supplementary Materials

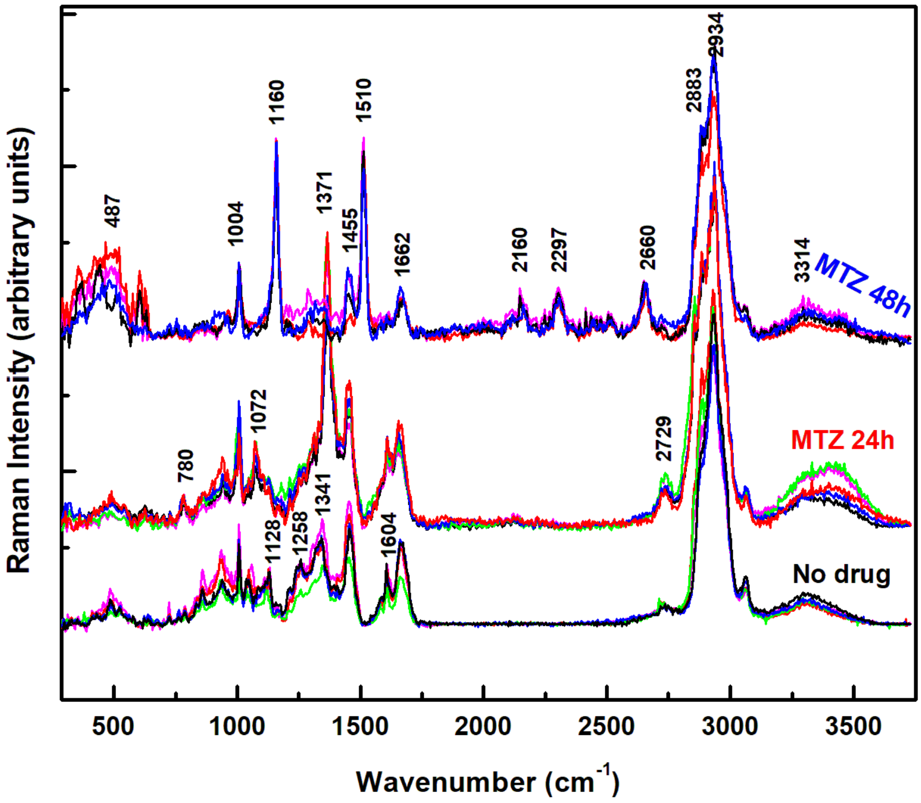

Figure S1. Integrated Raman spectra **from different cell images for the untreated and MTZ-treated for 24 h and 48 h (each spectrum averaging 10,000 spectra acquired per image).**

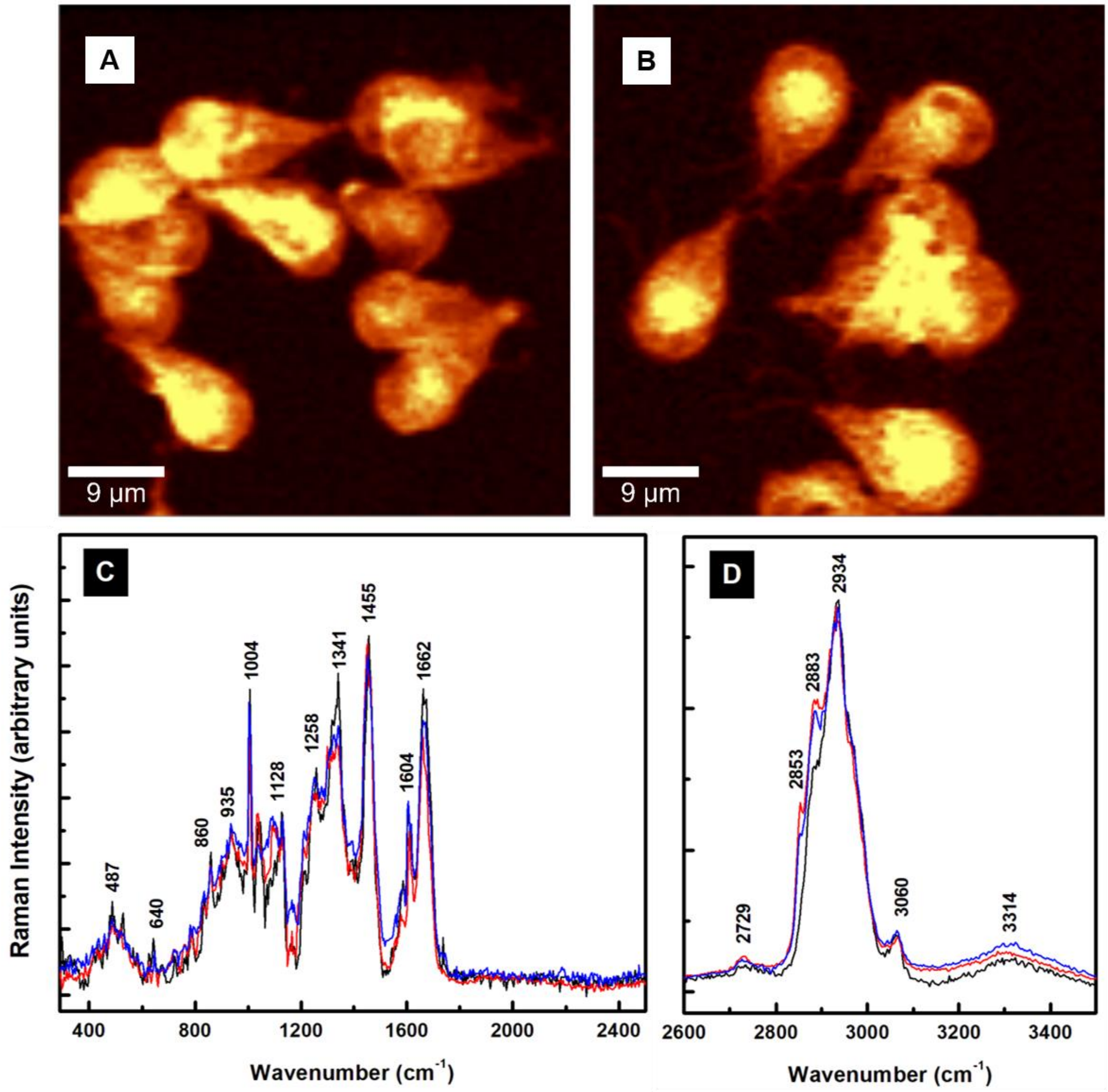

Figure S2. DMSO-treated *Giardia* cells: (**A**,**B**) Confocal Raman images of cells treated for 24h and 48h, respectively. (**C**,**D**) Integrated Raman spectra for **24h (red line) and 48h (blue line) of treatment**. The integrated spectrum (black line) of untreated cells is also plotted for reference.

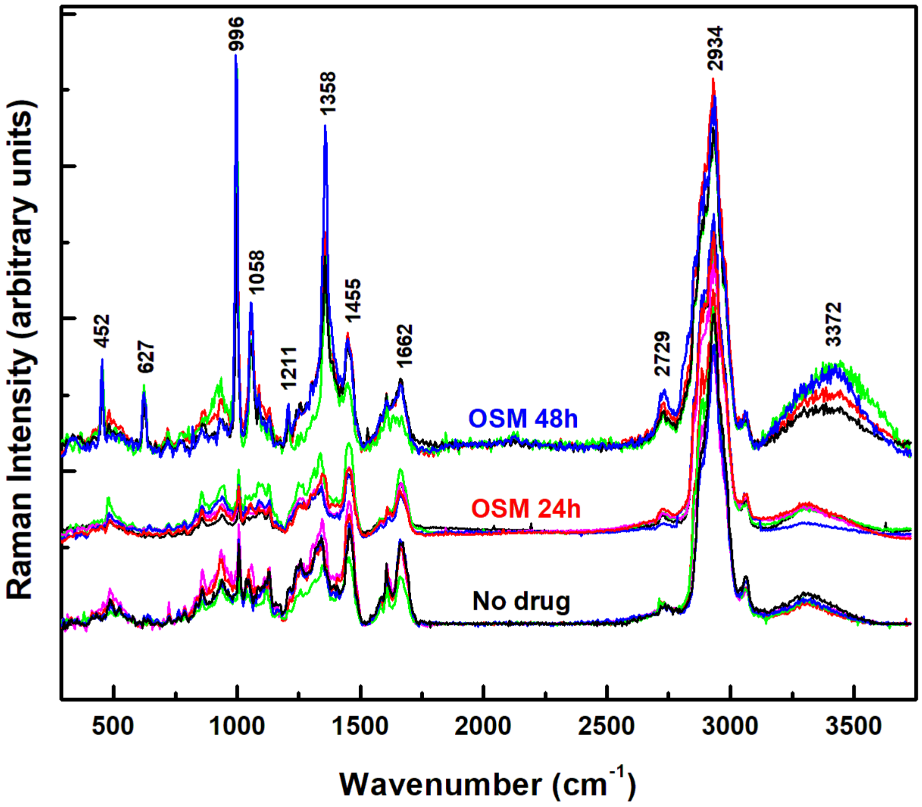

Figure S3. Integrated Raman spectra **from different cell images for the untreated and OSM-treated for 24h and 48h (each spectrum averaging 10,000 spectra acquired per image)**.
